# The psychological context of quality of life: a psychometric analysis of a novel idiographic measure of bladder cancer patients' personal goals and concerns prior to surgery

**DOI:** 10.1186/1477-7525-9-10

**Published:** 2011-02-15

**Authors:** Bradley Andrew Morganstern, Bernard Bochner, Guido Dalbagni, Ahmad Shabsigh, Bruce Rapkin

**Affiliations:** 1 Albert Einstein College of Medicine of Yeshiva University, 1300 Morris Park Avenue, Bronx, New York, 10461, USA; 2 Department of Urology and the Genitourinary Oncology Service, Sidney Kimmel Center for Prostate & Urologic Cancers, Memorial Sloan- Kettering Cancer Center, New York, New York, USA; 3 Department of Urology, Ohio State's Center for Advanced Robotic Surgery, Ohio State University Medical Center, Columbus, OH, USA; 4 Department of Epidemiology and Population Health, Albert Einstein College of Medicine of Yeshiva University, Bronx, New York, USA

## Abstract

**Background:**

Over the past two decades, there has been an increasing focus on quality of life outcomes in urological diseases. Patient-reported outcomes research has relied on structured assessments that constrain interpretation of the impact of disease and treatments. In this study, we present content analysis and psychometric evaluation of the Quality of Life Appraisal Profile. Our evaluation of this measure is a prelude to a prospective comparison of quality of life outcomes of reconstructive procedures after cystectomy.

**Methods:**

Fifty patients with bladder cancer were interviewed prior to surgery using the Quality of Life Appraisal Profile. Patients also completed the EORTC QLQ-C30 and demographics. Analysis included content coding of personal goal statements generated by the Appraisal Profile, examination of the relationship of goal attainment to content, and association of goal-based measures with QLQ-C30 scales.

**Results:**

Patients reported an average of 10 personal goals, reflecting motivational themes of achievement, problem solving, avoidance of problems, maintaining desired circumstances, letting go of roles and responsibilities, acceptance of undesirable situations, and attaining milestones. 503 goal statements were coded using 40 different content categories. Progress toward goal attainment was positively correlated with relationships and activities goals, but negatively correlated with health concerns. Associations among goal measures provided evidence for construct validity. Goal content also differed according to age, gender, employment, and marital status, lending further support for construct validity. QLQ-C30 functioning and symptom scales were correlated with goal content, but not with progress toward goal attainment, suggesting that patients may calibrate progress ratings relative to their specific goals. Alternately, progress may reflect a unique aspect of quality of life untapped by more standard scales.

**Conclusions:**

The Brief Quality of Life Appraisal Profile was associated with measures of motivation, goal content and progress, as well as relationships with demographic and standard quality of life measures. This measure identifies novel concerns and issues in treating patients with bladder cancer, necessary for a more comprehensive evaluations of their health-related quality of life.

## Background

Over the past two decades, there has been an increasing focus on quality of life outcomes in urological diseases. For most of this time, patient-reported outcomes research has relied on brief, structured assessments that constrain interpretation of the impact of disease and treatments on patients' quality of life. With the advent of alternatives for surgical reconstruction, bladder cancer patients face difficult choices and considerable uncertainty involving the long-term impact of treatment on lifestyle and activities. As such, it is necessary to understand treatment outcomes in light of particular patients' actual concerns and experiences. In this study, we introduce a new assessment procedure intended to provide perspective on individuals' priorities, based on Rapkin and Schwartz' (2004) quality of life appraisal model. This assessment is intended to complement standard measures of health-related quality of life (HRQOL), in order to distinguish differences in outcomes among patients with different priorities. In the present study we report results of a content analysis and psychometric evaluation of this Quality of Life Appraisal Profile, as prelude to a prospective comparison of outcomes of different reconstructive procedures for invasive bladder cancer.

HRQOL encompasses an array of patient-reported outcomes and can be defined as a patient's evaluation of the impact of a health condition and its treatment on all relevant aspects of life. HRQOL research on patients with bladder cancer is urgently needed, as bladder cancer is the fifth most commonly diagnosed cancer with an estimated 70,980 new cases in 2009 in the USA alone. It is the second most common malignancy of the genitourinary tract. It is the fourth most common malignancy in males, and the 11th in females [1]. Patients treated with radical surgery undergo one of three options for urinary diversions; ileal conduit urinary diversion, neobladder, or continent reservoir. The decision of the type of urinary diversion depends on surgeons experience and patient's performance status and comorbidities, in addition to patient's preference. Each of these reconstructive techniques carries different benefits and risks, and requires patients to exercise different self care skills.

QOL studies will help to fully articulate the impact of the different treatment modalities beyond survival rates, define levels of intervention in daily patient care, and point to factors that most significantly affect QOL in this specific setting (e.g., by selecting the optimal form of urinary diversion in the individual case or identifying a subset of patients who are likely to benefit in their adjustment by psychosocial intervention). In addition, bladder cancer is ranked as the most expensive common adult cancer in average health care costs incurred per patient from diagnosis to death in the United States. Thus, quality of life impacts are an important consideration in evaluating comparative effectiveness of treatments and controlling quality in these times of increasing costs, resource constraints, and priority setting within the health care market [2].

Existing quality of life measures provide a starting point for describing patients' experience of illness, treatment, and survivorship. Available standard assessments ask patients to rate their general quality of life in several domains (physical and emotional well-being, pain and symptoms, activities of daily living, role functioning, social activity, overall health) [3,4]. There are also standard rating scales to cover domains and problems of specific concern to bladder cancer patients: urinary, bowel, and sexual functioning [5-8]. More recently, scales have been introduced to examine treatment-specific questions such as adaptation to stomata, incorporation of lifestyle, activity or dietary changes, and stress and anxiety associated with watchful waiting [9,2,11]. Such rating scales are widely accepted and used, and considerable normative data are available, especially pertaining to general aspects of cancer-related quality of life and functioning [12].

Generic or global HRQOL instruments are applied across different diseases, conditions, populations, and concepts. Examples of generic instruments are the SF-36, the Quality of Well-Being scale, and the Sickness Impact Profile (SIP) [13-15]. Each of these instruments has undergone extensive developmental testing and is considered a valid measure. However, these measures may lack the specificity and sensitivity needed to capture relevant changes related to HRQOL specific to cancer patients [16].

Cancer-specific instruments attempt to measure HRQOL as it pertains to cancer treatment. This specificity leads to more narrowly-focused sets of items that may make them less likely to detect unanticipated effects. Two examples of cancer-specific measures are the European Organization for Research and Treatment of Cancer-QOL (EORTC QLQ-C30) and the Functional Assessment of Cancer Therapy general form (FACT-G) [17,3]. These cancer-specific measures also include cancer site- or treatment- specific modules to further address unique concerns. Currently, there are few site-specific instruments that measure HRQOL in bladder cancer patients who require cystectomy and urinary diversion. Two examples are the FACT-BL and the EORTC QLQ-BLM30 (the latter is still under development). In 2003, Cookson reported the results of an initial validation study of a bladder cancer-specific instrument, the Vanderbilt cystectomy index (FACT-VCI) [9].

Over the past two decades there have been several major studies comparing HRQOL for patients undergoing radical cystectomy and urinary diversion. However, these studies were limited in several significant ways. In a selective review of 15 more recent studies by Porter and Penson, ten used some type of previously validated health related HRQOL instrument and five used bladder cancer disease specific instruments only, five used general instruments only (not specific to bladder cancer), and five studies used a combination. However, only one of the bladder cancer disease specific instruments was validated in previous studies (FACT-Bl). These instruments were administered at different time points after surgery, usually in a single mailing or a single session in the clinic after radical cystectomy. Only one of the studies included a pre-operative baseline evaluation.[2]

Based on findings from general QOL instruments, most of these studies concluded that patients who have recovered from a radical cystectomy generally report a high HRQOL, not significantly different than people of similar age in the general population. Thus, despite the magnitude of the surgery and the degree of change in a major organ function, patients who recover from surgery and are cured appear to maintain or regain their HRQOL [18]. However, it is possible that these general measures are not sensitive to issues of unique importance to bladder cancer patients. More specifically, serious limitations affect the utility of standard HRQOL measures for understanding adaptation to bladder cancer. These limitations can be summarized in three ways:

Lack of coverage of key domains of concerns: Concise scales designed to be used in a wide variety of situations may miss important aspects of well-being or adjustment among certain patients or during certain periods of illness and recovery. For example, although patients may rate that they have little problem with urinary frequency, information may be missing about restrictions of activity, difficulties with use of pads, or occasional embarrassing situations that are having great impact on their quality of life.

Individual differences in experiences, values, and priorities: Ratings of standard scales do not take into account people's different interpretations and frames of reference. Such cognitive differences in appraising QOL may lead people to respond to items in ways that markedly affects the comparability of their responses. For example, when asked to rate difficulty in functioning, some individuals will compare their current status to past performance; others will make comparisons with other patients; still others will base ratings on what they expected or imagined when they heard the term "cancer". These individual differences may even enter into the meaning and interpretation of items: "satisfaction with role functioning" means something very different for people who want or expect to return to work versus people who want or expect to scale back. Comparison of individual differences in quality of life ratings requires some understanding of the personal context and meaning underlying these ratings, especially in heterogeneous patient populations.

Intra-individual "response shifts" in the appraisal of quality of life measures: Over the course of life-threatening illness, individuals may change their perspectives and expectations regarding quality of life. Such changes in perspective affect in turn the measurement of changes in HRQOL, including changes associated with the benefits of treatment. For example, immediately after completion of treatment, patients may base their ratings of fatigue and energy level on the immediate circumstances of their illness and recovery. Several months later, ratings of the same scales may reflect shifting expectations for greater stamina and a desire to return to a "normal" level of activity. Energy may have improved markedly, but changing expectations may mask this improvement or even indicate a decline.

These problems in measurement are not insurmountable. Rapkin and colleagues have developed a number of measurement approaches intended to complement standard measures of quality of life by eliciting differences and changes in patients' concerns, standards of comparison, and priorities for quality of life [19-22]. These "idiographic" (i.e., self-written) measures provide a way to gain better understanding of the meaning that patients impart to different scales, and to take this information into account in the evaluation of effects associated with illness and treatment. The rational behind this measure stems from the theory that comparable measures of aspirations (i.e. goals) are needed to fully evaluate individual's personal views of wellbeing. To achieve these ends, the goals elucidated must be compared in terms of the individual's own ideas of life satisfaction [23]. The model was recently validated in a sample of cancer survivors by Bloem and colleagues [24]. Li and Rapkin (2009) report an analysis of the association of changes in personal goals and changes in quality of life, among people living with HIV/AIDS, using idiographic data from the HIV Choices in Care Study [25]. They found that both positive and negative changes in quality of life ratings as well as apparent stability of ratings are each related to several distinct patterns of change in quality of life appraisal, even after controlling for demographic variables, baseline quality of life, and ensuing changes in clinical status and treatment.

Other previous studies have included idiographic measures of HRQOL, most notably, the Schedule for the Evaluation of Individual Quality of Life (SEIQOL). The SEIQOL is designed to capture between three and eight "cues" that fall in one of the generally agreed upon QOL domains of CASPER model - Cognitive, Affective, Social, Physical, Ecological and Religious domains. The respondent then rates his or her satisfaction with current functioning in regard to each personal statement on a 0-10 scale; and, then the relative importance of each cue is explored by having the individual rank his or her expected enjoyment of 30-50 hypothetical individual cases [26]. However, this approach relies heavily on individuals' interpretation of CASPER domains. Further, this approach is limited to a maximum of eight responses. We used the Rapkin and Schwartz (2004) approach to permit a wider variety and number of responses to emerge, and to allow individuals to directly evaluate their progress toward attainment of each identified personal goal [22].

This paper will describe the characteristics of the Quality of Life Appraisal Profile and discuss our system for coding patients' personal goals and motivational themes. We will then examine preliminary psychometric properties and validity of the QOL Appraisal Profile in the bladder cancer population, including associations with patient demographic and standard HRQOL measures. This descriptive measurement study is intended to set the stage for using this measurement in a large, prospective evaluation of changes in HRQOL associated with different surgical reconstruction procedures following radical cystectomy for bladder cancer.

## Materials and methods

### Sample

A sample of Memorial Sloan Kettering Cancer Center patients with bladder cancer were identified in the clinic and approached for participation. The Eligibility criteria included: patients diagnosed with bladder cancer and with the therapeutic plan of radical cystectomy; able to speak English; can provide informed consent; at least 18 years of age; may receive neoadjuvant or adjuvant chemotherapy; and may have had intravesicle chemotherapy or immunotherapy. Patients were excluded if they had metastatic disease at diagnosis or follow-up care was not obtained at Memorial Sloan Kettering.

Eligible patients who consented to the study were then scheduled for an interview prior to surgery, conducted by trained research assistants using the baseline version of the Quality of Life Appraisal Profile. Interviews were administered during scheduled sessions by telephone. The pace of the interview was guided by the respondent's ability to participate in the session. The other measures were filled out by the patient prior to surgery and mailed back. The current analysis includes the 50 baseline assessments.

### Measures

#### Brief Quality of Life Appraisal Profile

The Brief Quality of Life Appraisal Profile used in this study is adapted directly from measures developed by Rapkin and colleagues, including precursor versions of this same measure and the earlier Idiographic Assessment of Functional Status [21,22]. Briefly, this interview is designed to elicit patients' salient current concerns and goals, determine the activities that patients are pursuing to attain those goals, and then ask patients to rate the level of difficulty they are experiencing with each goal-attainment activity, their difficulty and need for support for reaching their goals, and overall distance from goal attainment. Thus, this procedure asks patients to generate their own sets of goal and activity statements that are each subsequently rated to obtain quantitative measures of distance from goal attainment, difficulty with key activities and adequacy of available support.

In order to elicit information on personal goals and concerns, we asked individuals to respond to seven probes, representing different motivational orientations: achievement, problem resolution, prevention or avoidance of problems, maintenance or keeping situations as they are know, acceptance of circumstances, disengagement from roles and responsibilities, and reaching important life events or milestones. For example, to probe achievement motivation, respondents are asked: "*In order to have the most satisfying life possible, what are the main things that you want to accomplish?*" For each probe, respondents are asked to provide up to three current goals. Thus, this assessment can elicit up to 21 different personal goals per respondent at the time of measurement. In order to facilitate subsequent scoring, interviewers are trained to clarify responses in several simple ways. First, when responses include the word "and", interviewers determine whether the statement constitutes one or several distinct concerns. Second, after the respondent has explained a concern, interviewers asked them to restate or summarize the concern in their own words, as a goal statement that completes the sentence, "I want to ..." These goal statements are recorded verbatim for content coding. Thus, this measure generates two types of data in addition to the overall number of goals:

**1**. The content of goals: As we shall detail below, we developed procedures to code multiple attributes of each goal statement, including life domains (e.g., work, family), interpersonal aspects, motivational themes (e.g., achievement, maintenance, disengagement), developmental themes (e.g., identity, intimacy), and cancer-relevance. Content codes summarized across an individual's goals identify that person's range and variety of concerns (for example, how many health concerns, how many family concerns, how many problems to solve, etc.).

2. Progress toward goal attainment. In addition to examining the content of cancer patients' personal goals, we were also interested in patients' appraisal that they were able to attain their goals. The assessment of successful goal attainment is distinct from personal goal content; that is, regardless of whether a goal involves health, family or employment, a patient can rate their sense of progress toward fulfillment [27]. Goal attainment was ascertained by reading back each of the patients' goal statements and then asking, "On a scale from 0 to 10, how much progress have you made toward reaching this goal? 0 means no progress at all and 10 means you are completely successful in reaching this goal." These ratings may be readily combined (e.g., average rating, minimum rating) across all of an individuals' goal statements.

#### EORTC QLQ-C30

The European Organization for Research and Treatment of Cancer QLQ-C30 (QLQ-C30) version 3.0 is a well-validated instrument that is designed to give a broad assessment of health-related quality of life in cancer patients. It was developed by the EORTC's Study Group on Quality of Life whose mandate is to develop an integrated measurement system for evaluating the QOL of cancer patients participating in international clinical trials. There are 30 items in this instrument which evaluate 6 major domains: physical, role, emotion, social, cognition and a global assessment of quality of life. In addition, three symptom scales are used to measure fatigue, pain, emesis; and six single items assess financial impact, dyspnea, sleep disturbance, appetite, diarrhea and constipation. This core instrument covers a general range of quality of life issues relevant to all patients with cancer. It is designed to be supplemented with more disease specific modules, which can assess aspects of QOL of particular importance to various patient subgroups [17,28].

#### Demographic and Health History Measures

Standard questions used in the clinic at the time of physician visit, plus chart information were used to gather information on patients' gender, race/ethnicity, education, marital/partner status, employment status, religious participation and affiliation, disease and treatment history, and co-morbidities.

### Analysis Plan

Our analysis included examination of patient characteristics and quality of life, thematic content coding of responses to the Brief Quality of Life Appraisal Profile, examination of the relationship of goal attainment to goal content codes, and association of goal based measures with QLQ-C30 sub-scales. Due to the limited sample size, we report results at the p < .10 two-tailed level of significance.

### Analytic Software

All responses were first recorded with paper and pen. Subsequently the responses were entered into to a Microsoft Access database. Qualitative responses were imported to a Microsoft Excel file for coding. The analyses were completed in SPSS version 17.0.

### Human Subjects Protections

The study was reviewed by the Memorial Sloan-Kettering Cancer Center IRB under the protocol number 08-076. All patients provided written informed consent and HIPAA authorization documents.

## Results

### Patient Characteristics

Patients were predominately male (68%) with mean age of 66 years old (standard deviation = 10 years). The majority of patients were Caucasian, with two African American patients and one Hispanic patient. The educational level was high, with 51% patients attaining a bachelors degree or higher. Approximately 74% of patients reported being married and 40% reported being employed at least part time. Six of the patients had received neoadjuvant chemotherapy prior to the interview, including two who were still receiving chemotherapy at the time of the interview. Fifteen patients were within the window of bowel preparation (approximately two days before the surgery date) for surgery. These clinical variables were taken into account in analyzing patient responses to idiographic and standard HRQOL measures.

### Completeness of Data

Due to the complex nature of treating patients with bladder cancer, the difficult course leading up to the surgery, and the method of retrieval of the survey, several items were missing at the time of data collection. Goal data were obtained by interview, and thus were complete for 50 patients. However, three individuals did not complete the demographics form and could not be reached prior to analyses. An attempt was made to determine missing demographics data through chart review for these three individuals. In addition, a total of 37 out of 50 patients were included in this analysis had returned the QLQ-C30. Comparisons of demographic and goal characteristics for full cohort versus the 37 patients who returned QLQ-C30 demonstrated only minor differences.

### Coding of Goal Statements

Overall, goal probes yielded 503 individual goal statements across 50 respondents, an average of 10.06 goal statements per respondent. Of these responses, 22.7% were elicited by the achievement motivation probe, 15.5% by the problem solving probe, 14.3% by the problem prevention/avoidance probe, 17.7% by the maintenance probe, 3.6% by the disengagement probe, 9.3% by the acceptance probe, and 16.9% by the milestones probe. Several statements (n = 6) were coded as non-answers and hence not included in the averaged coded percentages. (See Table 1)

**Table 1:** Goal content categories, average Kappas, % usage out of 503 goal statements, and % of 50 patients with ≥ one response/code

Goal Content Codes by Thematic Categories	Average Kappa^‡^	Proportion of All Responses (N = 503)	% Patients Responding in Category (N = 50)
**MOTIVATIONAL THEMES**	0.926	103.39%	100%

Achievement	0.856	24.65%	98%

Problem Solving	0.918	15.31%	98%

Prevent or Avoid Problems	0.930	13.72%	88%

Maintenance/Keeping Things as They Are Now	0.957	19.09%	92%

Letting Go of Responsibilities or Tasks	0.961	3.78%	34%

Accepting Circumstances	0.940	9.74%	76%

Reaching an Event/Milestone	0.920	17.10%	92%

**HEALTH and TREATMENT**	0.7	77.73%	100%

Cancer-Specific Goals	0.829	29.82%	92%

Provider and Treatment Related Concerns	0.717	26.24%	94%

Health Issues - Not Cancer	0.659	18.29%	82%

Existential and End of Life Concerns	0.595	3.38%	28%

**FUNCTIONING**	0.722	22.47%	62%

Independent Functioning*	0.374	13.12%	60%

Continence/Stoma	0.891	7.36%	40%

Sexual Functioning	0.902	1.99%	14%

**MENTAL HEALTH and PERCEPTIONS**	0.318	11.72%	56%

Mental Health and Mood State	0.498	7.75%	52%

Personality and Personal Attributes*	0.362	2.98%	22%

Body Image *	0.095	0.99%	10%

**COMFORT and LIFESTYLE**	0.542	52.09	100%

A Life on My Own Terms*	0.305	13.52%	72%

Leisure Activities	0.708	12.33%	66%

Living Comfortable*	0.195	9.74%	58%

Return to Life Before Cancer	0.480	7.36%	54%

Travel	0.900	5.96%	46%

Sports	0.881	2.78%	24%

Exercise*	0.326	0.40%	4%

**EVENTS**	0.509	11.92%	64%

Major Milestone	0.617	4.97%	38%

Special Plans	0.526	4.37%	34%

Cyclic Events*	0.385	2.58%	22%

**RESPONSIBLITIES**	0.743	19.48%	76%

Work and Unemployment	0.802	8.15%	52%

Financial Concerns	0.808	7.75%	50%

Education	0.891	1.79%	16%

Accomplishing Chores and Tasks*	0.472	1.79%	16%

**RELATIONSHIPS and FAMILY CONCERNS**	0.782	37.98%	78%

Interpersonal Relationships	0.716	16.50%	70%

Family In General	0.775	8.95%	56%

Children/Grandchildren	0.874	5.77%	40%

Intimacy and Sexual Relationship Concerns	0.611	3.78%	30%

Friends in General	0.933	2.98%	26%

**HOME and COMMUNITY**	0.725	7.16%	36%

Living Situation, Housing, Neighborhood	0.886	3.98%	28%

Societal and Altruistic Concerns*	0.504	1.79%	12%

Community Involvement and Voluntarism	0.643	0.99%	6%

Religious and Spiritual Concerns	0.866	0.40%	4%

**PROBLEMS and CONFLICTS**	0.655	5.98%	34%

Conflicts*	0.192	3.38%	30%

Drug and Alcohol Use	0.773	0.60%	6%

Legal and Crime Concerns	1.000	0.20%	2%

Racism^		0.00%	0%

Immigration and Citizenship^		0.00%	0%

**OTHER**	0.53	1.59%	12%

Non-answer*	0.194	1.19%	10%

Fantasy	0.866	0.40%	4%

* Arbitrated category

^ Not used due limited responses in the category coded by the raters

‡ Average kappa values prior to arbitration

In order to analyze cancer patients' verbatim goal statements, it was necessary to develop variables that described the issues and themes patients mention. This is known as thematic analysis or content analysis. Our content analysis involved a three-stage process:

### Step 1

As a preliminary step we started with codes developed for our earlier HIV Choices in Care Study [25,29]. These 26 codes included issues related to health, family, life span development, work roles, emotional well-being and the like. We changed specific references to "HIV/AIDS" to "bladder cancer," and also added two codes involving common concerns that were not represented in the HIV study, continence/stoma, and sexual functioning. This yielded a total of 28 codes.

### Step 2

We wanted to be sure that our coding system was exhaustive, in order to represent the full range of concerns of cancer patients facing surgery. To accomplish this, we assigned three judges the task of examining the 503 goals statements. Judges were asked to independently sort their goals into homogeneous categories, with the sole criterion being that statements placed in a category must be similar in all important ways. Judges were asked to record the "goal attributes" that they used to make distinctions among categories. Judges first conducted a gross sort, based on major life domains (e.g., family, health, tasks), and then sorted within major categories to identify further distinctions. Thus, goal categories could be determined by two or more life domains. For example, the goal statement, "I want to take care of my grandkids" differs from "I want to have strength to take care of grandkids" because of the latter's added focus on personal functional capacity. This step of coding was complete when judges had fully sorted all goals into homogenous categories, and had identified all distinctions among categories. From this initial sorting a subsequent 12 more codes were established yielding a total of 40 goal codes including the major motivational themes.

A few explanations of examples of how goal coding decisions were made will help to clarify our methods:

1. The coders would score goal statements as a cancer specific goals (29.82%) if it fit the definition: Anything that has to specifically do with cancer such as urination problems, bleeding, pain, in reference to the cancer or "current problem." This definition intends capture the physical symptoms associated with the disease process experienced by the individuals. It is noteworthy that such a large percentage of the patients expressed statements concerning this particular goal when asked open-ended questions. Three examples of examples when patients were asked the achieve probe are: 1) "urinate normal;" 2) "to go to the bathroom without pain;" and 3) "surgery to be complete and a success." These statements conceptually range from basic bodily functions to the complex idea that surgical success is defined solely by the individual own interpretation of the idea and ability to understand fully the possible treatment outcomes and side effects.

2. It is also illustrative to examine examples quotes defined as a major motivational theme as it demonstrates yet another dimension of the QOL Appraisal Profile and further helps elucidate the nuances of the profile. The major theme of reaching an event/milestone (17.10%) is defined as: Any activity, milestone, completion of a project or activity (i.e., graduation, retirement) the person wishes to reach; events should generally involve reaching a certain date or point in time. Three of examples of such statement when the patient were asked that specific probe are: 1) " [I] would like to make it to [my] father's age of 92;" 2) "see grandkids grow up;" and 3) "sleep through the night." Here the examples demonstrate breadth of the individuals own view point and interpretation of the probe; however, the profile and it's coding process still manages to capture the theme. All three quotes are representations of the individual's own idea of what they focus on and deem as an obvious/important events in their lives. The coding process continues to clarify the goal statement by erring on the side of commission when scoring the statement as the other content categories (i.e. "see grandkids grow up" was also coded as interpersonal relationships and children/grandchildren as well).

### Step 3

We also needed to account for the fact that some responses reflected motivational themes that differed from the eliciting probe. For example, an individual might say that they wanted to "accomplish" avoiding further illness or reaching a milestone. Thus, we had judges determine the appropriate motivational themes associated with each response, and used these codes for subsequent analysis.

### Step 4

Once these codes were compiled, three coders were trained to independently go back to apply these codes to the 503 statements. Thus, all goal statements were coded according to 7 motivational themes as well as one or more of these 40 content categories.

In order to evaluate the quality of our coding system, we calculated inter-coder reliability using kappa coefficients for each of the 47 goal attributes [30]. Kappa coefficients represent agreement corrected for chance overlap due to category prevalence. Kappa can range from -1 to 1, with 0 representing agreement no greater than chance. If kappa for a given code was high, it meant that raters tended to strongly agree in assignment of codes. If kappa was low, it meant that there was disagreement among judges regarding use of a particular code, so decisions involving that code required arbitration. Thus, if the average kappa between pairs of raters was below 0.4 (considered to be a moderate level of agreement), coders reviewed all responses assigned that code by any rater. This procedure ensured that there was considerable agreement about all final codes used to describe the goal statements.

Goal codes are reported in Table 1, including original kappas (averaged across all pairs of judges) prior to arbitration, the percent of the 503 statements assigned each code, and the percent of 50 patients reporting at least one goal in a particular category. The goal coding categories are grouped according to overarching domains, including the Motivational Themes (7 categories); Health and Treatment (4 categories); Functioning (3 categories); Mental Health and Perceptions (3 categories); Comfort and Lifestyle (7 categories); Events (3 categories); Responsibilities (4 categories); Relationship and Family (5 categories); Home and Community (4 categories); Problems and Conflicts (5 categories); and Other/Non-Answer (2 categories).

On average, 2.46 content codes and one motivational theme were applied to each goal statement. These codes could occur in any combination - for example, wanting to avoid health problems impacting the family relationships, or wanting to reduce distress by learning to accept the loss of work roles. Note that for 17 responses (3.38%), coders identified more than one motivational theme associated with a response. Thus, the total number of motivational themes codes adds up to slightly more than 100%.

There were several noteworthy patterns in goal content, evident in the middle column of numbers on Table 1, based on number of responses (503). The highest percentage of all goal statements pertained to Health and Treatment themes. On average, 29.82% of the 503 goal statements mentioned by patients were cancer related concerns. The second most frequently mentioned theme pertained to provider and treatment related concerns at 26.24%. Many patients mentioned non-cancer health concerns (18.29%). Comfort and lifestyle also tended to be coded frequently, including a life on my own terms (13.63%), leisure activities (12.33%), and living comfortably (9.74%). Interpersonal relationships (16.50%) and goals pertaining to patients' family in general (8.95%) were also common.

It is also worthwhile to compare the second column of numbers on Table 1, indicating proportion of all responses, to the third column that summarizes the proportion of 50 patients who gave at least one response in a given category. Although many codes occurred less frequently out of 503, they still pertain to a high proportion of patients. For example, although only 3.78% of the 503 goals statement involved the motivational theme of disengagement/letting go of responsibilities and roles, 34% of the 50 patients mentioned at least one goal in this domain. In regards to understanding differences among people in terms of their goal content, this measure of disengagement may be more important than codes that occur with high frequency categories. For example, almost all patients reported achievement (98%) and problem solving (98%) goals, indicating relatively little variation in use of these categories. Many of the goal content codes demonstrated good variability at the individual level, including existential and end of life concerns (mentioned at least once by 28% of 50 patients), independence functioning (60%), continence and stoma (40%), mental health and mood state (52%), travel (46%), financial concerns (50%), and work and unemployment (52%). Some important categories were mentioned by relatively few patients, such as sexual functioning (14%), body image (10%) and social and altruistic concerns (12%). Despite this low prevalence, we decided to retain these categories in order to identify patients with special concerns or unique issues related to QOL.

### Associations among Motivational Themes and Goal Content

In order to better understand individuals' patterns of response to the idiographic measure, we examined the association of the 40 goal content dimensions with the seven motivational themes. By chance, we would expect 28 significant correlations at p < .10. In fact, 40 significant correlations were evident at this level. These relationships indicated that certain areas of concern were more involved with achievement, others with acceptance and still others with problem solving. For example, individuals who mentioned a higher proportion of concerns related to cancer also tended to mention a higher proportion of goals associated with coming to accept one's situation (r = .25) and a lower proportion of goals associated with active achievement (r = -.33). Overall, concerns associated with cancer were generally correlated with lower levels of motivation to actively address problems. Specifically, lower levels of achievement motivation were also associated with concerns about independence functioning (r = -.30), continence/stoma (r = -.29), provider and treatment issues (r = -.27), and existential/end of life matters (r = -.27). In addition, the desire to return to life prior to cancer was associated with higher problem solving motivation (r = .27).

Contrary to this cancer-specific pattern, bladder cancer patients who mentioned a higher proportion of health concerns that were not related to cancer tended to express significantly greater levels of both achievement motivation (r = .36) and problem solving motivation (r = .37). The proportion of mental health and mood state related concerns was also associated with achievement (r = .50) and problem solving (r = .37). Individuals reporting higher proportion of concerns related to drug and alcohol use tend to report greater motivation for prevention and avoidance of problems (r = .30) as well as orientation toward achievement (r = .50) and problem solving (r = .30). This pattern of findings suggests that at this point in time, prior to surgery, a number of bladder cancer patients were focused on learning to accept cancer, but nevertheless wanted to solve problems that kept them from returning to their life prior to cancer diagnosis. At the same time, these patients were much more actively engaged with overcoming other health, mental health and substance use concerns.

Differences in motivational themes were also associated with interpersonal relationships and specific activities. For example, a greater proportion of goals involving the family was associated with a higher levels of motivation to keep things as they are now (r = .35) and as well as the desire to reach significant milestones (r = .37). As would be expected, the desire to reach milestones was also associated with mention of special plans (r = .33), cyclic events like holidays (r = .43), and travel (r = .29).

### Progress toward Goal Attainment

Our assessment scheme made it possible to examine perceived progress toward goal attainment associated with different types of goals. We examined correlations of 50 individuals' average progress ratings with the proportion of individuals' goal statements coded with each motivational theme and goal content codes. By chance, we would expect 5 significant correlations at p < .10. We observed 12 correlations significant at this level. Here again the profile demonstrates, even with such few subjects, its ability to capture subtle nuances important to the patients and further develops a construct for understanding the patients' goals, which in turn help to define their QOL.

In terms of motivational themes, patients with a higher proportion of maintenance goals reported greater progress toward goal attainment (r = .38). Alternatively, a higher proportion of problem prevention/avoidance goals was associated with lower perceived progress (r = -.32). In terms of goal content, individuals reporting a higher proportion of health and functioning goals indicated less progress toward goal attainment, including cancer-specific goals (r = -.25), concerns about independent functioning (r = -.35), goals related to living comfortably (r = -.36) and concerns related to continence and living with a stoma (r = -.29). Here again making logical sense in a pre-treatment cohort as they are focusing on attaining good health or avoiding undesired side effects rather than already attaining or experiencing them, respectively. Lower progress was also associated with greater proportion of goals pertaining to mental health and mood state (r = -.24) and to drug and alcohol use (r = -.29). Alternatively, greater progress was reported by individuals with a high proportion of family-related goals (r = .47) and more general interpersonal concerns (r = .25). Greater progress toward goals was also reported by individuals who were looking forward to cyclical events like holidays or anniversaries (r = .26) or who mentioned involvement with sports (r = .28). These examples help to define and construct an understanding of the population being studied in ways that would be expected and hence will be the baseline for future analysis.

We were also interested in whether patients tended to be more optimistic or pessimistic in general, when rating progress on their various goals. To examine this, we calculated the intraclass correlation, a measure of the proportion of variance associated with between-patient differences in progress ratings. Indeed, the intraclass correlation of the progress goal rating was quite high at 0.45. This intraclass correlation supports the use of the within-patient average rating of progress as a measure of patients' general experience of goal attainment. Thus, average level of progress toward attaining one's own personal goals provides a psychometrically sound and theoretically grounded idiographic measure of quality of life.

### Analysis of the Construct Validity of the Brief QOL Appraisal Profile

A key concept we used to examine the psychometric properties of the Appraisal Profile is construct validity. Construct validity pertains to all types of measurement [31]. In the present case, our assessment purports to elicit an accurate representation of an individual's personal goals. We are using a thematic coding system to derive measures of individuals' goal content. Although the content of an individual's responses may have face validity, it is nonetheless necessary to determine whether the indices we derived by summing up codes of these responses behave in a manner consistent with our interpretations. In terms of convergent validity, a key aspect of construct validity, we expect that the content of individuals' goals will tend to correspond with aspects of their life circumstances. Thus, we expect indices of work-related goal content to be higher among individuals who are employed; more goals related to specific health and functional problems among people who are sicker; and more goals related to care giving and family well-being among people with young children. We also expect goal to differ in line with normative social roles regarding gender and age. Although these correlations may seem obvious, it is still useful to report this sort of convergence, just as it would be useful to show that a depression scale distinguishes people who have a clinical diagnosis from those who do not. In both cases, we would be concerned about the validity of assessment if these distinctions among known groups were not observed. [20,22]

In terms of discriminate validity, a second key aspect of construct validity, the theory underlying the quality of life appraisal model explicitly suggests that the criteria individuals use to evaluate their quality of life ought to be substantially independent from their level of quality of life. In general, it is possible for people who emphasize domains of family, work or spiritual growth report either high or low levels of quality of life. Indeed, the appraisal model predicts that individual differences in appraisal criteria (e.g., goal content) will affect the correlates of QOL ratings, but not their overall levels [22]. Of course, there must be caveats to this global prediction - for example, some concerns are unequivocally negative (coping with disease recurrence; attempting to solve medical, financial or family problems, coming to terms with the death of a loved one) and would be associated with worse QOL by virtue of precipitating life events. Thus, we expect that measures of QOL will be uncorrelated with goal content codes except in cases where goal content reflects unambiguously negative life events. (Goals associated with desired life events are more complicated because individuals with serious illness may have mixed emotions about these e.g., "I want to be healthy enough to be at my daughter's wedding"). [20,22]

### Demographic correlations with Codes and Progress Goal Ratings

For the next step of our analysis, we examined the correlation of seven motivational themes, forty goal content codes, the number of goals and progress toward goal attainment with five demographic measures (age, gender, marital status, education level, and employment). Given the size of this analysis, we would expect 25 significant correlations by chance at p <.10 level. This set of analyses yielded 34 significant correlations between goal measures and demographics, which are reported in Table 2. For sake of space, we only included goal measures on this table that had at least one significant correlation with a demographic measure. It is noteworthy that there were no demographic differences in the proportion of goals associated with the various motivational themes. However, there were differences evident in overall number of goals, goal content, and progress toward goal attainment. We will briefly summarize significant differences in goals associated with each demographic measure. Last, it is important to recognize that within these demographic analyses convergent validity yet again emerges independently. (Note that judges were completely blind to demographic characteristics of respondents when rating goal statements).

**Table 2:** Significant correlations between goal measures and demographics (N = 50)

	Age	Female	Married	Level of Education	Any Work
**NUMBER of GOALS**			0.35	0.27	0.34

					

**HEALTH and TREATMENT**					

Health Issues - Not Cancer					-0.32

**FUNCTIONING**					

Sexual Functioning				0.29	0.23

**MENTAL HEALTH and PERCEPTIONS**					

Mental Health and Mood State	-0.30	0.37			

Personality and Person Attributes			0.26		

Body Image			-0.31		

**COMFORT and LIFESTYLE**					

A Life on My Own Terms		0.35			

Leisure Activities	0.47				

Living Comfortable			-0.34		

Return to Life Before Cancer	-0.32	0.26			

Sports	0.25	-0.28			

Exercise	-0.28				0.25

**RESPONSIBILITIES**					

Work and Unemployment	-0.32				0.41

Financial Concerns		-0.33			

Accomplishing Chores and Tasks		0.33			

**RELATIONSHIPS and FAMILY CONCERNS**					

Family in General			0.30	-0.28	

Intimacy and Sexual Relationship Concerns	-0.35				0.24

**HOME and COMMUNITY**					

Living Situation, Housing, Neighborhood					-0.30

Societal and Altruistic Concerns	0.24				

**PROBLEMS and CONFLICTS**					

Conflicts			-0.25		-0.29

					

**AVERAGE PROGRESS TOWARD GOAL ATTAINMENT**	0.25	-0.35		-0.24	

Age: Older respondents tended to report higher proportions of goals related to leisure activities, sports, and social altruistic concerns. Alternatively, younger respondents reported a higher proportion of concerns related to intimacy and sexuality, work, mental health and mood state, and exercise. This pattern seems consistent with an interpretation that younger adults experience bladder cancer diagnosis as a more disruptive, off-time event than their older counterparts. Consistent with this interpretation, older adults tended to rate higher average progress toward goal attainment.

Gender: Women tended to report a higher proportion of mental health and mood-related concerns as well as the desire to live life on one's own terms and to try to return to their life before cancer. Alternatively, men tended to focus more on accomplishing financial concerns, accomplishing chores and tasks, and sports. These findings seem consistent with well-established differences in gender norms. (Note that men also reported significantly higher average progress toward goal attainment.)

Marital Status: Married individuals and those with significant partner relationships tended to report a greater overall number of goals than unmarried. Married individuals also had a greater proportion of goals related to family, as well as goals about one's own personality and body image. Unmarried individuals reported significantly more goals related to body image, addressing interpersonal conflicts, and living comfortably.

Education: More educated individuals in our sample reported a greater number of goals, and proportionately more goals associated with concerns about sexual functioning. Individuals with lower levels of education had a greater proportion of concerns about family. Lower education was also associated with greater perceived progress toward goal attainment.

Employment: Employment status was significantly correlated with patients' age (r = -.29). As such, there were some similarities in the patterns of correlations of goal measures with employment and age. As might be expected, employed individuals had a higher proportion of goals associated with work and unemployment, intimacy and sexual relationships, sexual functioning, and getting exercise. Individuals who were not employed reported proportionately more concerns about health problems unrelated to cancer, their living situations including housing and neighborhood issues, and interpersonal conflicts. Employed individuals also reported a greater number of goals.

### Correlations between QLQ-C30 Quality of Life Ratings and Measures of Personal Goals

As a final step in our analysis, we examined the association of variables derived from the idiographic Brief Quality of Life Appraisal Profile and ratings on QLQ-C30 quality scales. When compared with published QLQ-C30 norms based on 23,556 patients with all cancers at all stages, the pre-surgical bladder cancer patients in our sample reported significantly better quality of life on most QLQ-C30 scales with the exception of emotional functioning, social functioning, fatigue, nausea and vomiting, and financial status, where there were no differences. Similarly, in comparison with the QLQ-C30 reference sample of 246 urological cancer patients, respondents in our sample reported better quality of life in all areas except emotional functioning, nausea and vomiting, and financial status.

We examined correlations among the 15 QLQ-C30 scales to determine whether any were so highly correlated as to be redundant. Although there were many significant correlations among the EORCT scales, their magnitudes were only moderately high. Out of 105 correlations, the majority (61%) were between r = -.20 and .20. Only 9% of correlations among pairs of QLQ-C30 scales had an absolute value greater than r = .50 and no correlation exceeded an absolute value of r = .70. Given the relative independence of these QLQ-C30 scales and limited options for data reduction with our small sample size, we decided to examine simple bivariate correlations between QLQ-C30 and other measures.

There were relatively few correlations between the five demographic measures and 15 QLQ-C30 scales. By chance, we would expect 8 correlations to be significant at p < .10, and indeed we observed 8 significant correlations. Thus, these associations must be considered spurious and are not reported. There was almost no association between QLQ-C30 scales and the absolute number of personal goals reported. The only significant correlate was an association between greater reported social functioning and fewer goals. This difference in number of goals may reflect individuals struggling to maintain social ties and activities. This correlation may represent the individual trying out a number of different directions.

In our analysis of the associations among QLQ-C30 scales with motivational themes and goal content codes, we would expect 66 correlations to be significant at p < .10 level by chance alone. In fact, we observed only 53 significant correlations, fewer than would be expected by chance alone. This is consistent with our overall discriminant validity expectation that goal content should be largely independent of quality of life ratings. However, many of the correlations that were significant were actually quite large in absolute magnitude, with 34 significant at p < .05. This is one more than would be expected by chance alone. Although this result is marginal, we decided to report relationships of the QLQ-C30 with motivation and goal content measures because of the sizable correlations that did occur. These relationships help to shed light on more nuanced relationships between various goal content domains. Results of this analysis are reported in Tables 3 and 4 at the p < .05 level. For sake of space, we only report on those goal measures that were significantly correlated with at least one of the QLQ-C30 scales.

**Table 3:** Correlations between QLQ-C30 Functional Scales with Motivational Themes and Goal Content (N = 37)

	Global QOL	Physical Function	Role Function	Emotional Function	Cognitive function
**Motivational Themes**					
Let go	**-0.36**	**-0.46**	**-0.46**		
Accept				**0.33**	
**Mental Health and Perceptions**					
Mental Health and Mood State	**-0.39**				

**Responsibilities**					
Work and Unemployment			**-0.38**		
Education					**0.34**
Accomplishing Chores & Tasks					**0.34**

**Relationship &Family Concerns**					
Interpersonal Relationships				**-0.41**	
**Home and Community**					
Societal and Altruistic Concerns				**0.32**	
Religious and Spiritual Concerns					**-0.36**

**Problems and Conflicts**					
Drug and Alcohol Use				**-0.38**	

Note:

Correlations included on this table are significant at p < .05 (two-tailed);

Goal measures that had no significant correlations with QLQ-C30 Scales were omitted from this table.

**Table 4:** Correlations between QLQ-C30 Symptom Scales with Motivational Themes and Goal Content (N = 50)

	Fatigue	Nausea/Vomiting	Pain	Insomnia	Appetite	Constipation	Diarrhea	Financial
**Motivational Themes**								
Achieve								**-0.40**
Solve				**-0.37**				
Let go					**0.50**		**0.49**	
Accept							**0.49**	

**Comfort and Lifestyle**								
Leisure Activities		**0.40**						
Sports	**0.37**	**0.37**						

**Events**								
Major Milestone						**0.32**		
Special Plans	**0.34**							

**Responsibilities**								
Financial Concerns				**0.33**				
Education	**0.35**		**0.41**					

**Relationship &Family Concerns**								
Interpersonal Relationships				**0.33**				
Family in General				**0.34**				
Children/Grandchildren							**0.42**	
Friends in General	**0.47**			**0.38**				

**Problems and Conflicts**								
Drug and Alcohol Use				**0.33**				**0.47**

Note:

Correlations included on this table are significant at p < .05 (two-tailed); Goal measures that had no significant correlations with QLQ-C30 Scales were omitted from this table.

#### Association of Goal Content Dimensions with QLQ-C30 Global and Functioning Scales

As is evident in Table 3, individuals with diminished global, physical, and role functioning tended to report higher levels of disengagement or "letting go" goals. In terms of the QLQ-C30 functioning scales, emotional and cognitive functioning showed the strongest and most consistent associations with the content of bladder cancer patients' goals. Prevalent concerns about drugs and alcohol and about interpersonal relationships were associated with worse emotional functioning, while societal and altruistic concerns was correlated with better emotional functioning. A greater number of acceptance goals were correlated with greater QLQ-C30 emotional functioning. In addition, higher cognitive functioning was reported by those patients more concerned with education and accomplishing chores and tasks. Alternatively, self-reported cognitive functioning was lower among those patients with greater religious and spiritual concerns. Individuals with more work and employment related concerns tended to report worse role functioning.

#### Association of Goal Content Dimensions with focal QLQ-C30 Symptom Scales

In regard to motivational themes, letting go was associated with greater reported problems with appetite and diarrhea. Diarrhea was also associated with motivation to come to accept one's situation. Individuals with higher levels of achievement motivation report fewer financial problems, while those focused on solving problems indicate lower levels of insomnia. In terms of goal content, greater prevalence of symptoms was associated with increased concerns related to comfort and lifestyle, responsibilities, and relationship conflicts. It is possible that the salience of certain symptoms was heightened among individuals with certain concerns. For example, greater fatigue was reported by individuals concerned about sports, special plans, educational goals, or family relationships. Problems with insomnia were greater for individuals concerned about friends, family and other interpersonal relationships, drug and alcohol use and finances. Ratings of financial difficulty were greater among those with drug and alcohol problems. Other strong associations may reflect ways in which symptoms can disrupt goal attainment: Thus, greater problems with nausea and vomiting were associated with patients concerns about leisure activities and sports, while reported pain was associated with educational goals. Constipation and diarrhea were greater among those concerned about reaching milestones or being with grandchildren, respectively.

#### Progress toward Personal Goal Attainment

It is noteworthy that there were no significant correlations between progress toward goal attainment and any of the QLQ-C30 scales. This may be due to restriction of range of the QLQ-C30 (given the relatively high levels of functioning in our sample). Alternatively, idiographic measures of goal attainment may tap into aspects of quality of life not represented on traditional quality of life rating scales.

## Discussion

This purpose of this paper was to provide psychometric information on the performance and correlates of the Brief Quality of Life Appraisal Profile idiographic assessments in a sample of bladder cancer patients scheduled for cystectomy. Our analyses were intended to show the utility of this assessment procedure, characterize the types of information that it yields, and provide correlations with patient demographics and with QLQ-C30 scales. These correlations speak directly to the construct validity of the goal-related measures derived from the idiographic assessment. That is, we want to observe whether measures of goals behave in ways that are consistent with theoretical expectations about associations among these measures, and with external variables. In this vein, there were a number of major findings and conclusions that we can draw from this study:

### Feasibility of Assessing Quality of Life Using the Brief Appraisal Profile

Based on responses to the idiographic measure, it is clear that bladder cancer patients recruited to this study were willing and able to complete this measure. This is consistent with findings by Rapkin and colleagues in earlier studies of people living with HIV/AIDS [19,21,25]. The bladder cancer patients in this sample were more than 20 years older than participants in these earlier studies. Further, unlike this earlier work, interviews here represent the first time that the idiographic Brief Quality of Life Appraisal Profile has been used to provide a true pre-treatment baseline. Patients who agreed to interview encountered no difficulty in responding to this measure, even during this stressful period of time

Patient responses to this measure were high quality. All but 6 out of 503 separate verbatim responses to the seven probes could be scored as goal statements. This suggests that our interview procedures for eliciting and refining patient responses worked as planned.

There was considerable variability in the number and nature of responses across patients. Number of goal statements ranged from 4 to 16, with a mean of approximately 10. Patients responded to all seven of the probes. Our coding procedures demonstrated that responses to probes tended to be consistent with the motivational theme induced by that probe (e.g., when we asked about problem solving goals, maintenance, or disengagement, patients tended to respond accordingly).

### Adaptation of Assessment and Scoring Procedures for Bladder Cancer Patients

In this study, we added a new motivational probe focused on reaching milestones that was not included in earlier versions of the appraisal Profile. All but 4 of 50 patients provided at least one response to this probe. Reaching and participating in milestones and major life cycle events accounted for 17.1% of the 503 goal statements, generated after patients had responded to the first six probes. This indicates that it is an important way to elicit patients' concerns that ought to be included in this assessment scheme.

The coding system that we developed retained almost all 28 categories used in Rapkin and colleagues' earlier work on people living with HIV/AIDS. The three categories out of 28 not used by patients in this sample were ones that were clearly more relevant to individuals living with HIV/AIDS such as discrimination and stigma. Twelve categories were added, reflecting concerns specific to bladder cancer.

Judges were able to apply codes with a relatively high level of consistency and inter-rater reliability (kappa). However, it was necessary to arbitrate many categories, particularly those that appeared with low frequency. Based on this initial coding experience, it will be possible to provide a detailed manual for future judges, including rules and examples pertaining to low frequency codes.

### Evidence for the Construct Validity of Goal-Based Measures

Patterns of association between motivational themes and goal content codes made sense from a psychological perspective. For example, among these newly diagnosed cancer patients, goals, goals related to cancer were more likely to be elicited by the probe asking what they were trying to accept. Alternatively, non-cancer related health goals were associated with achievement motivation and problem solving. This pattern likely reflects the timing of this baseline assessment relative to their diagnosis of cancer. This pattern reflects a correspondence between individuals' current life circumstances and the content of their personal goals, and so supports the validity of our measurement. As these individuals go forward with surgery and recovery, we would expect that they would start to report cancer-related problems and achievement-related goals, similar to their non-cancer health problems.

In addition, personal goal measures were also associated with demographic measures in ways that were logical and consistent, supporting convergent validity. Age related-differences in content strongly suggest that younger patients were experiencing the bladder cancer diagnosis as a more disruptive, off-time event. Gender differences were also evident, with women focusing more on emotional and expressive goals, while men emphasized tasks and tangible impacts. Married patients identified more family and intimacy concerns, while unmarried patients mentioned issues of body image. Workers indicated greater concerns about employment as well as goals consistent with their younger age.

As expected, there were relatively few significant correlations of QLQ-C30 scales with motivational themes and goal content adding some support to our expectation of discriminate validity. It is important to emphasize the importance of this finding from the perspective of the QOL appraisal model of Rapkin and Schwartz (2004). If there were many high correlations between measures of appraisal and measures of level of quality of life, it would suggest that the appraisal model is moot (e.g., that individuals with similar levels of QOL tend to report similar concerns, and hence there is no reason to worry about direct comparability of QOL scores). In fact, the relative independence of these domains supports our major contention that appraisal measurement offers a way to provide an independent, interpretive frame for understanding people's ratings of QOL.

Several correlations between goal content measures and QLQ-C30 scales did emerge. To some extent, these were consistent with our expectation that correlations would reflect unambiguously negative life experiences such as poor health or personal losses. However, additional patterns also emerged that shed light on how content of personal goals might influence appraisal of functioning and symptoms.

Individuals expressing concerns pertaining to interpersonal relationships and to addiction expressed the worst emotional functioning. Alternatively, those focused on more altruistic concerns reported better emotional status. Although the causal direction of this relationship is not clear, it makes sense that individuals with better emotional well-being are able to extend their concerns to others. Schwartz and colleagues have reported on the importance of altruism in helping individuals maintain their quality of life, and this relationship supports that observation [32].

Level of cognitive functioning showed an interesting pattern of associations with the content of individual's concerns. People who's goals involved further education or accomplishing tasks reported higher levels of cognitive functioning, while those reporting greater spiritual concerns indicated lower levels of cognitive functioning. Again, although the causal direction is uncertain in this cross-sectional study, the correlation is provocative. It may be that people who are experiencing cognitive problems have necessarily had to reduce their involvement with education or managing their own affairs. They may also place a greater premium on spiritual and religious means of coping. Alternatively, those who maintain challenging educational or practical concerns may be unwilling to admit declines in cognitive functioning. Longitudinal research including actual measures of performance and independence will help to clarify this pattern.

It is also noteworthy that individuals who reported more work and employment related concerns tended to report worse role functioning. This relationship in particular demonstrates the important of taking individuals' personal criteria into account when evaluating quality of life. Individuals who rate role functioning items with work performance in mind are necessarily using different performance criteria and have access to different feedback than those who are not focused on work. Note that this relationship does not depend solely upon whether an individual works, but whether work is a focus of their concerns.

Correlations of QLQ-C30 with symptom measures suggested several possible mechanisms that might be at play. On the one hand, the presence of certain concerns may amplify negative responses to symptom measures; for example, people concerned about interpersonal relationships, special plans, leisure activities or education reported heighten levels of fatigue. Whether being tired is disruptive of these activities, or engaging in these activities is tiring, the relationship seems to reflect a direct influence. On the other hand, symptoms like pain, constipation and nausea are not likely to be caused by individuals' activities, so observed relationships probably represent actual or feared disruptions of efforts to attain desired personal goals.

### Lack of Relationship between QLQ-C30 Scales and Average Progress toward Goal Attainment

Progress toward goal attainment proved to have high internal consistency, as evidenced by a high intraclass correlation. That is, whether optimistic or pessimistic, individuals tended to have similar perceptions of progress toward all of their different goals. Progress was associated with maintenance goals as well as goals associated with family and desired activity. Health and mental health concerns were correlated with lower levels of perceived progress.

One unexpected finding was the lack of relationship between progress toward goal attainment and any of the QLQ-C30 scales. Progress toward goal attainment can be understood as a direct measure of quality of life consistent with theoretical definitions of quality of life as the correspondence between perceived and preferred circumstances (i.e., how far are you from having things the way you want?). Lack of correlation between this goal-based measure and QLQ-C30 scales may have occurred for several reasons. First, our sample of pre-surgical bladder cancer patients demonstrated higher levels of quality of life and less variance than EORTC normative samples. Thus, lack of correlation may be due to a relative restriction of range on the QLQ-C30 scales. Greater variation on these ratings might emerge over the course of treatment and recovery. If so, correlations between functional and symptom measures and progress toward goal attainment may emerge.

Second, attenuated correlations between QLQ-C30 scales and progress toward goal attainment could be due to differences in quality of life appraisal and response shift. It may be that goals and concerns reported by patients have already been adjusted, selected or censored based on individuals' beliefs about what they can or should be trying to accomplish. Simply put, it may be that people with diminished functioning tend to consider less demanding or ambitious goals, while those with higher levels of functioning mention goals that are in some way harder to achieve. If so, ratings of progress toward goal attainment would be calibrated against these different metrics. With a larger sample to permit multivariate analysis, our assessment strategy will allow us to determine whether differences in goal content serve to reduce correlations between measures of progress and patient-reported functioning and symptoms. Even more interesting, prospective data will allow us to determine whether changes in functioning systematically lead to less demanding or challenging personal goals, and subsequent recalibration of ratings of progress toward goal attainment.

Third, it may be that progress toward goal attainment is truly distinct and independent from functioning and symptom measures. It is true that correlations among different quality of life measures are often quite high. However, this may reflect similar item content and method variance. The idiographic measure of progress toward goal attainment is derived in a way that is completely different from standard quality of life scales like the QLQ-C30, and may represent an alternative perspective on quality of life. In future research, it will be necessary to examine whether goal attainment relates to external criterion measures, such as community adjustment, performance measures, independent living and the like. For example, in a study of people living with HIV/AIDS Rapkin and colleagues found that idiographic ratings of functional status complemented standard quality of life rating scales in accounting for physician-rated performance and other criteria[21]. It would be useful to replicate these analyses for bladder cancer patients.

### Study Limitations

Due to the small sample size (N = 50), power examine certain correlations is limited. However, even with this smaller size, many important correlations and observations consistent with expectations and common sense clearly emerged.

Several additional issues arose during data collection due to the timing of the interviews. On a number of occasions, the interview would be completed during the patients' bowel preparations for the surgery. This procedure can tend to be difficult for patients and may influence response. To accommodate this, interviewees were allowed to take breaks as needed. Other patients completed the Quality of Life Appraisal Profile and other measures during the time that they were receiving neo-adjuvant chemotherapy. In order to see whether these confounders affected our results in any systematic way, analyses were rerun including only those individuals who had not yet started bowel preparations or started treated at the time of the interview. There were no significant changes in the results based on this analysis. However, affects may emerge in large samples.

Several additional limitations exist in the current data. The first is the lack of ethnic variation in the sample population. The current sample includes primarily Caucasian patients, which could limit the external validity. However, as enrollment increases the diversity will most likely increase. Another consideration is the possibility that we will need to include additional goals codes after surgery and during recovery. If so, additional codes can be readily incorporated into our coding schema using procedures outlined herein.

Finally, despite our best efforts to minimize time demands and increase comfort, the extent of assessment required for this measure may be too arduous for some participants. It will be worthwhile to develop more parsimonious measures of goal content and distance from goal attainment.

### Future Issues

This research combines both qualitative and quantitative methodologies to facilitate better understanding of patient concerns. Examining patients in this manner will contribute to a more comprehensive quantitative understanding of bladder cancer patients' HRQOL by eliciting novel information not traditionally captured by standard measures. Such research could support the creation of decision aids for patients undergoing radical cystectomy. Such decision aids would take into account individual differences in priorities and concerns that affect treatment outcomes. Assessment procedures such as the one presented here can also serve as a vehicle to improve patient-provider communication by helping to elicit and clarify topics of greatest importance to patients.

## Conclusion

Even with a small sample size, the Brief Quality of Life Appraisal Profile demonstrates many noteworthy and meaningful correlations among various measure of motivational themes, goal content and progress toward goal attainment, as well relationships with demographic and standard health-related quality of life measures. These relationships suggest the psychometric soundness and construct validity of measures derived from this assessment procedure. Further research is needed to clarify causal relationships. This measure will serve to identify novel concerns and issues in treating patients with bladder cancer, as we undertake more comprehensive evaluations of their health-related quality of life.

## Competing interests

The authors declare that they do not have competing interests.

## Authors' contributions

BAM assisted in the organization/administration of the study, gathering of the data, analysis of data, and drafting of the publication. BB and GD helped to develop the study, write the protocol, recruit patients, and write the introduction of the publication. AS helped to develop the study, write the protocol, and write the introduction of the publication. BR developed the instrument, analysis of data, helped to develop the study, write the protocol, and write the publication. All parties have received the manuscript and have reviewed it.
